# Linking Psychotic‐Like Experiences and Brain White Matter Microstructure in Young Women

**DOI:** 10.1002/brb3.70587

**Published:** 2025-05-30

**Authors:** Rikka Kjelkenes, Sara Fernandez‐Cabello, Irene Voldsbekk, Madelene Christin Holm Bukhari, Andreas Dahl, Ingvild Sandø Lofthus, Henning Stople Rise, Christian K. Tamnes, Ivan I. Maximov, Lars T. Westlye

**Affiliations:** ^1^ Department of Psychology University of Oslo Oslo Norway; ^2^ Center for Precision Psychiatry, Division of Mental Health and Addiction Oslo University Hospital Oslo Norway; ^3^ Division of Mental Health and Substance Abuse Diakonhjemmet Hospital Oslo Norway; ^4^ PROMENTA Research Center, Department of Psychology University of Oslo Oslo Norway; ^5^ Department of Health and Functioning Western Norway University of Applied Sciences Bergen Norway; ^6^ KG Jebsen Centre for Neurodevelopmental Disorders University of Oslo Oslo Norway

## Abstract

**Background:**

Psychotic‐like experiences (PLE) represent a risk factor for later psychotic disorders and a marker for general mental distress. The connectivity and microstructure of key brain white matter pathways, including fronto–temporal trajectories, have been implicated in psychosis and psychosis‐risk. Although sex differences in PLE prevalence and characteristics have been reported, most neuroimaging studies of PLE have primarily included mixed‐sex, samples and little research has been carried out in females only. This study examines the associations between PLE and white matter characteristics in young to middle‐aged females.

**Methods:**

We analyzed cross‐sectional diffusion magnetic resonance imaging (dMRI) and self‐reported data from 661 females aged 9–44 years using the 15‐item version of The Community Assessment of Psychic Experiences (CAPE) questionnaire. Associations between CAPE subscales and other psychopathology measures were tested. Using linked independent component analysis (LICA), we decomposed the voxel‐wise data from 24 dMRI metrics across five different diffusion models into 10 spatially independent components. We then examined the association between the LICA subject weights and age. Next, we tested for associations between the LICA subject weights and both CAPE total and subscales scores using Bayesian statistics.

**Results:**

PLE were broadly associated with various domains of psychopathology and psychosocial factors. Moderate evidence emerged for an association between PLE and an LICA component reflecting a broad and complex pattern of diffusivity in major pathways, including the inferior fronto– occipital fasciculus, anterior thalamic radiation, and forceps minor. The persecutory ideations subscale showed the strongest evidence of an effect.

**Conclusion:**

PLE in young females are associated with a distinct multimodal white matter pattern reflecting microstructural characteristics in key commissural, association, and thalamocortical pathways in young females. The findings support that LICA is a valuable tool for fusing and decomposing advanced dMRI metrics to delineate white matter patterns that show sensitivity to PLE and mental distress.

## Graphical abstract

FLICA was used to decompose 24 diffusion metrics into 10 independent components. Associations between subject weights on the different components and symptoms of psychotic‐like experiences were tested for using Bayesian statistics. We found evidence of an association between one component (LICA 07) and PLE.

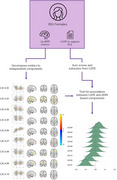

## 1 Introduction

Childhood psychotic‐like experiences (PLE) are risk factors for later psychotic disorders (Lindgren et al. 2022; Staines et al. 2022), with an estimated 4‐fold increased risk for developing a psychotic disorder later in life among those who reported PLE compared to those who did not (Healy et al. 2019; Isaksson et al. 2022). Due to the co‐occurrence of PLE with disorders such as substance abuse, depression, anxiety, and posttraumatic stress disorders (Bourgin et al. 2020; Kelleher et al. 2012; Varghese et al. 2011), PLE have been suggested as a severity marker of general mental illness and not only a psychosis risk factor (Stochl et al. 2015). A recent study exploring PLE in a population sample of > 21,000 Norwegian 14‐year‐olds found that 33% reported often experiencing one or more PLE (Birkenæs et al. 2024). Another study of 29,021 males reported that 26% had experienced at least one psychotic‐like episode (Birkenæs et al. 2023). These two studies highlight that PLE is common both in adolescents and adults (Birkenæs et al. 2023). Studies have also reported sex differences in PLE, with women showing higher prevalence and different expression with females reporting more hallucinations and persecutory ideation (PI) (Isaksson et al. 2022; Kråkvik et al. 2015; Stainton et al. 2021; Welham et al. 2009; Wu et al. 2022). These observed sex differences of PLEs might stem from a complex interplay of biological, developmental, and socio‐environmental factors, including hormonal influences, differences in coping mechanisms, and societal norms. However, the pathological mechanisms underlying these differences are unknown. The common occurrence in the population, the increased risk of psychotic disorders, the proposed links with other domains of psychopathology, and the reported sex differences suggest different pathological mechanisms between men and women.

Epidemiological and clinical studies document sharp increases in the incidence of mental disorders during adolescence and early adulthood (Kessler et al. 2007; Solmi et al. 2022). The emergence of mental health issues during adolescence and early adulthood suggests that neuroimaging markers reflecting neurodevelopmental processes in this period of life may be particularly relevant candidates for mapping the neural underpinnings of mental health problems (Solmi et al. 2022). Diffusion MRI (dMRI) studies have shown protracted development of myelinated white matter connections, typically displayed by increasing FA and decreases in MD, with especially fronto–temporal connections showing protracted development throughout adolescence and early adulthood (Krogsrud et al. 2016; Lebel et al. 2012, 2017), which has been hypothesized to be linked to the increased incidence of psychopathology during this period (Asato et al. 2010; Paus et al. 2008). A previous study, using multimodal fusion, found that the pattern most associated with general psychopathology and cognition involved frontotemporal connectivity and the uncinate fasciculus (Alnæs et al. 2018). Further, sex differences in maturational timing of white matter tracts have been reported, with females showing earlier maturation than males (Asato et al. 2010; Beck et al. 2023; Lawrence et al. 2023). Sex differences in brain maturation could potentially explain part of the observed sex differences in the emergence, prevalence, and expression of mental disorders (Dalsgaard et al. 2020; Rosen et al. 2020; Sommer et al. 2020).

dMRI is sensitive to the micro‐ and macrostructural architecture of human brain white matter. Mathematical and biophysical models for analyzing dMRI, such as diffusion tensor imaging (DTI, (Basser et al. 1994)), diffusion kurtosis imaging (DKI, (Jensen et al. 2005)), spherical mean techniques (SMT, (Kaden et al. 2016)), white matter tract integrity (WMTI, (Chung et al. 2018; Fieremans et al. 2011), and restriction spectrum imaging (RSI, (White et al. 2013)), provide complementary information about the underlying brain tissue, and when combined, may enable a comprehensive investigation of the complex biological mechanisms mediating the associations between psychopathology and brain white matter architecture.

The main aim of the current study was to determine the associations between PLE and white matter characteristics in a sample of young to middle‐aged females. To examine the association between PLEs and other domains of psychopathology, we also included questionnaires assessing a range of symptoms. To this end, a Norwegian convenience sample of 661 females aged 9–44 years completed online questionnaires assessing PLE and other mental health problems, including depression and anxiety, and key psychological and social factors, including social support, perceived stress, and sleep. We used FSL's linked independent component analysis (LICA) (Groves et al. 2011) to combine white matter metrics from various diffusion models, including DTI, DKI, RSI, SMT, multi‐compartment SMT, and WMTI. We tested for associations between the corresponding LICA subjects’ weights and total, as well as subtypes, of PLE using a Bayesian regression framework. Based on a previous study (Alnæs et al. 2018), we hypothesized that components reflecting delayed white matter development would be associated with PLE.

## 2 Methods and Materials

### 2.1 Participants

**FIGURE 1:**
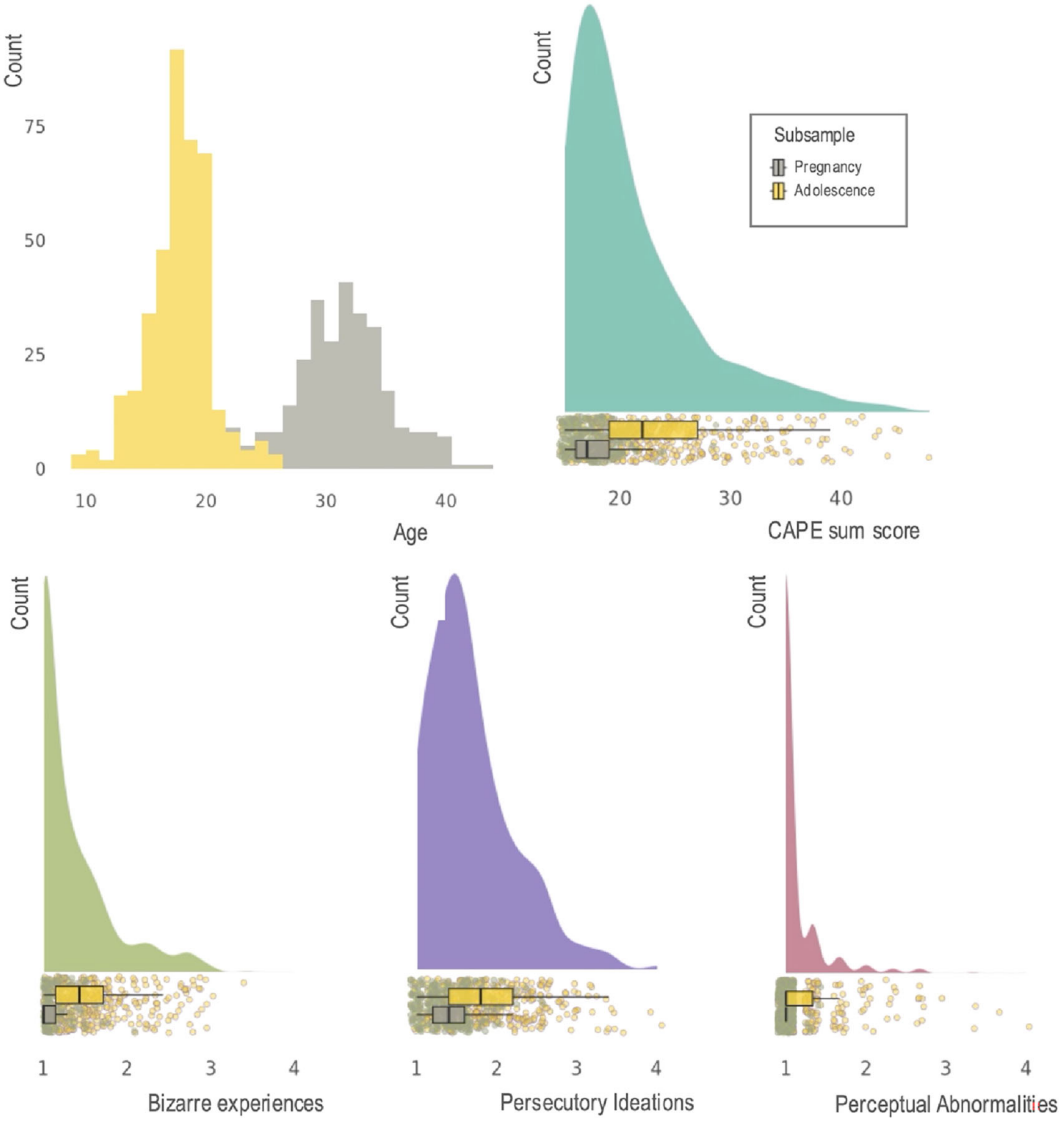
On the top left histogram showing the age distribution of the included adolescence subsample (yellow) and pre‐pregnancy subsample (grey) are presented. On the top right the distribution for the complete sample on total CAPE score is plotted on top of scatters showing individual scores and box plots showing group means. On the lower panel similar plots are shown for the CAPE subscales persecutory ideations, bizarre experiences and perceptual abnormalities. For the subscales average item score is used. CAPE = The Community Assessment of Psychic Experiences.

The participants were drawn from the ongoing Brains and Minds in Transition (BRAINMINT) study in Oslo, Norway. Figure 1 summarizes the demographic and key PLE measures for the sample. The current sample consisted of 661 females (9–44 years). Of the total sample, 270 participants (22–43 years) were recruited to be part of a longitudinal pregnancy study, following them before, during and after pregnancy. All included data from this sub‐group were collected before pregnancy. In addition, 391 participants (9–25 years) were part of the adolescence subsample. The only exclusion criteria were standardized MRI exclusion criteria (non‐removable metal, braces, claustrophobia). All scans were evaluated by a trained neuroradiologist, who assigned each scan a score of 1–4, with 1 indicating no findings and 4 indicating potential abnormal brain pathology. MRI was completed on 750 females, from these, 28 participants were given a score of 3 or 4 and were excluded from the analysis. After MRI quality control (see the details below), sufficient quality MRI data and questionnaire data were available for 661 participants.

The study was approved by the Regional Committee for Medical and Health Research Ethics, South‐East Norway. All participants, or legal guardians for children under the age of 16, provided informed consent prior to enrollment.

#### 2.1.1 Demographic, Medical, and Psychopathology Assessment

Participants completed online questionnaires including assessments of PLE, personality traits, perceived level of stress and social support, and symptoms of anxiety and depression within the 2 weeks following their MRI scan (see Figure S1 for distribution of summary measures of psychopathology and psychosocial adjustment).

To assess PLE, the 15‐item version of The Community Assessment of Psychic Experiences (CAPE, (Capra et al. 2013)) was used. The CAPE‐15 items can be categorized into three subscales: bizarre experiences (BE), perceptual anomalies (PA), and PI. We included the total score and the mean item score from each subscale in the analysis. For personality traits, we used a Norwegian 20‐item short version of the Big Five Inventory (BFI, John and Srivastava 1999). We used sum scores reflecting the major personality traits neuroticism, openness, extraversion, conscientiousness, and agreeableness. To measure the level of perceived stress, we used the sum score from the 10‐item version of the Perceived Stress Scale (PSS) (Cohen et al. 1983). For social support we used the Multidimensional Scale of Perceived Social Support (Zimet et al. 2010). We used the sum scores on the subscales friends and family, with high scores indicating more perceived support. To measure symptoms of generalized anxiety, we used the sum score on 7 items from the Patient Health Questionnaire (PHQ, Kroenke et al. 2010). We used the Edinburgh Postnatal Depression Scale (Cox et al. 1987, Cox et al. 1996) to measure symptoms of depression among women recruited to the pregnancy sub‐study, and the Short Mood and Feelings Questionnaire (Goodman 1997) for the younger group. These depression scales were harmonized by dividing the sum scores by the max total score, multiplied by 100.

### 2.2 MRI Acquisition

All participants were scanned at Oslo University Hospital on a 3T GE SIGNA Premier MRI scanner with a 48‐channel head coil. The MRI protocol included structural (T2 CUBE, T1 MPRAGE, T2 FLAIR CUBE), multiband resting‐state functional MRI, and multiband multi‐shell dMRI. Paddings were used to reduce head motion, and an extender was used for the participants with a bigger head. Participants listened to the radio during the scan.

dMRI data were acquired using a multiband (acceleration factor: 3) echo planar imaging (EPI) sequence with the following parameters: Repetition time = 4.75 s, echo time = 84 ms, flip angle = 90, field‐of‐view = 240 × 240 × 148 mm^3^, slice thickness = 1.7 mm, in‐plane resolution = 1.7 × 1.7 mm^2^, 104 volumes. 96 diffusion directions were included (*b*‐values: 500/6 dirs, 1000/15 dirs, 2000/15 dirs, 3000/60 dirs). The diffusion gradient scheme was adapted by the DISCOBALL algorithm (Stirnberg et al., 2009), with a scan time of 8:58 min. In addition, 7b  =  0 volumes with reversed phase‐encoding direction were acquired.

### 2.3 dMRI Processing

We used an optimized dMRI processing pipeline (Maximov et al. 2019), which included the following steps: 1) Noise correction using PCA of Marchenko‐Pastur (MP‐PCA) (Veraart et al. 2011; Veraart et al. 2013), 2) Gibbs ringing correction (Kellner et al. 2016), 3) estimation of EPI originated distortion, motion, and eddy‐current induced artefacts using top‐up and eddy in FSL (Andersson and Sotiropoulos 2016), 4) spatial smoothing using fslmaths from FSL with a Gaussian kernel of 1, and 5 mm^3^) diffusion metrics estimation. The following diffusion models were applied to compute a range of diffusion metrics for each scan: DKI (Jensen et al. 2005) including DTI, RSI (White et al. 2013), WMTI (Chung et al. 2018; Fieremans et al. 2011), and SMT (Kaden et al. 2016) (See Table S1 for details).

Before further analysis and processing, a rigorous quality control procedure was implemented. We ran the EDDY QC framework consisting of Quality Assessment for dMRI (QUAD) and Study‐Wise Quality Assessment for dMRI (SQUAD) (Bastiani et al. 2019). We flagged and manually checked datasets that were two standard deviations away from the mean on the various quality measures including motion, outliers and SNR/CNR. In total 68 participants were flagged, among which 33 were removed after manual evaluation of the image quality. This resulted in a final dMRI sample of 750 females, among whom 661 also had complete questionnaire data available.

We used Tract‐based spatial statistics (TBSS) for voxelwise analysis (Smith et al. 2006). TBSS projects all subjects’ fractional anisotropy (FA) data onto a mean FA skeleton. We aligned all maps that passed the quality control to the FMRIB58_FA template supplied by FSL, the binary skeleton was created by thresholding the FA skeleton with the value 0.2. We then applied the identical normalization and skeletonization steps on the non‐FA data.

### 2.4 Linked Independent Component Analysis

We used FMRIB's LICA (Groves et al. 2011, 2012) to decompose the 24 dMRI skeletonized metric maps into spatially independent modes of variance. LICA was run with 3000 iterations, and a final model order of 10 was heuristically chosen based on a large degree of modality fusion. We inspected all components’ spatial maps (24 spatial maps per component) and subject loadings for outliers, one component that was largely driven by one subject was discarded (the distribution of the subject weights for each component is shown in Figure S2).

### 2.5 Statistical Analysis

Statistical analyses were performed in R version 4.2.0 (R core Team 2020). To assess the relationship between CAPE total score, sub‐scales of CAPE, and other domains of psychopathology and psychosocial factors while also controlling for age partial Spearman correlations were calculated. Partial spearman correlation was chosen to address the non‐linearity of the data and allowed us to control for age, given its potential influence on the associations. For the remaining analyses we applied a Bayesian approach using the brms (Bürkner, 2017) package in R and examined linear associations between the subject weights of each of the LICA components and the CAPE total‐ and sub‐scale means. For all analyses we used a prior strongly centred around zero (mean = 0, SD = 0.5), reflecting our conservative assumption of minimal effect size. This choice helps regularize associations, favoring data‐driven inference and reducing false positives. Separate models were run for each LICA components to examine its relationship with each of the different CAPE scores. The different CAPE scores were included as the dependent variable, and age and LICA subject weights were entered as independent variables. Prior to analysis, age and CAPE scores were *z*‐scored. This centered the data around zero with a standard deviation of one, thereby enhancing interpretability and comparability without altering the underlying relationships between the variables.

The Savage–Dickey density ratio method was used to calculate the Bayes factors (BF) (Wagenmakers et al. 2010), which indicates the strength of evidence in favor of the null hypothesis or the alternative hypothesis. BF = 1 can be interpreted as evidence in either direction. The following values can be interpreted as weight towards the alternative hypothesis with the following strengths: 0.3–1 (*anecdotal*), 0.1–0.3 (*moderate*), 0.03–0.1 (*strong*), 0.01–0.03 (*very strong*), < 0.01 (*extreme*). BF > 1 provides evidence towards the null hypothesis: 1–3 (*anecdotal*), 3–10 (*moderate*), 10–30 (*strong*), 30–100 (*very strong*), >100 (*extreme*) (Wagenmakers et al. 2010).

## 3 Results

### 3.1 Associations Between Psychotic‐Like Experiences, Psychopathology and Psychosocial Factors

We examined the relationships among the different subscales of PLEs from the CAPE questionnaire using Spearman's rank correlation analyses. The results indicated strong correlations between PIs and BE (*r* = 0.52, *p* < 0.001) and moderate correlations between PIs and perceptual abnormalities (*r* = 0.36, *p* < 0.001), as well as between perceptual abnormalities and BE (*r* = 0.39, *p* < 0.001).

**FIGURE 2:**
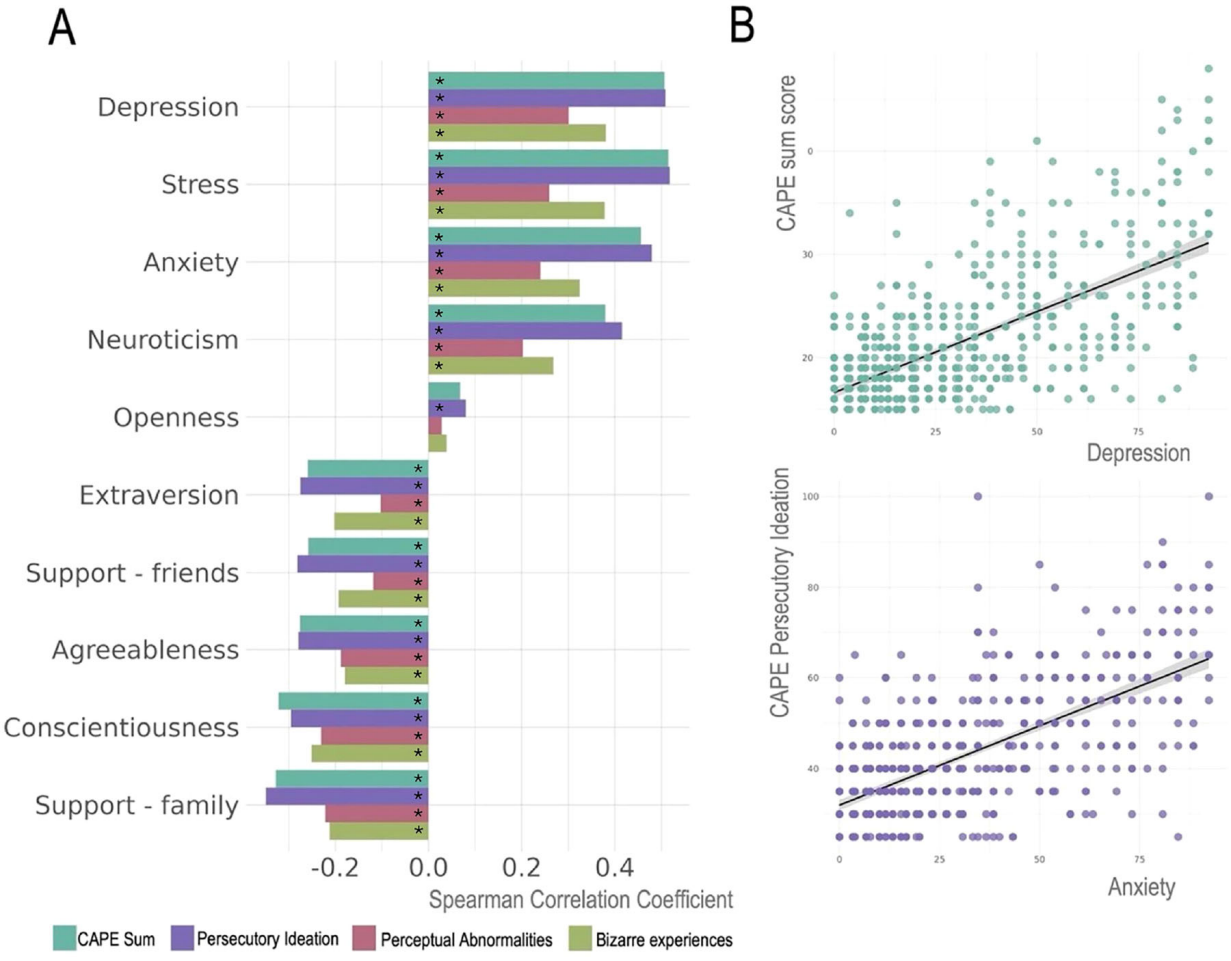
Correlations between CAPE and other domains of psychopathology and psychosocial factors. A) Barplot showing the Spearman partial correlations (corrected for age) for each of the CAPE subscales and select demographic, psychosocial and psychopathological measures. B) Scatterplots showing relationships between CAPE sum score and depression symptoms (top) and between persecutory ideation and anxiety symptoms (bottom). * = *p* value < 0.05.

Spearman's correlations revealed positive associations between CAPE scores and other clinical measures, with varying strengths across scales (Figure 2). Particularly strong associations were observed between PI and measures of stress (*r* = 0.52, *p* < 0.001), depression (*r* = 0.51, *p* < 0.001), and anxiety (*r* = 0.48, *p* < 0.001). The perceptual abnormalities subscale was the subscale that showed the weakest correlation to the other factors, with the strongest, yet modest, correlation being with depression (*r* = 0.30, *p* < 0.001).

### 3.2 Decomposition of dMRI Maps Using Linked Independent Component Analysis

**FIGURE 3:**
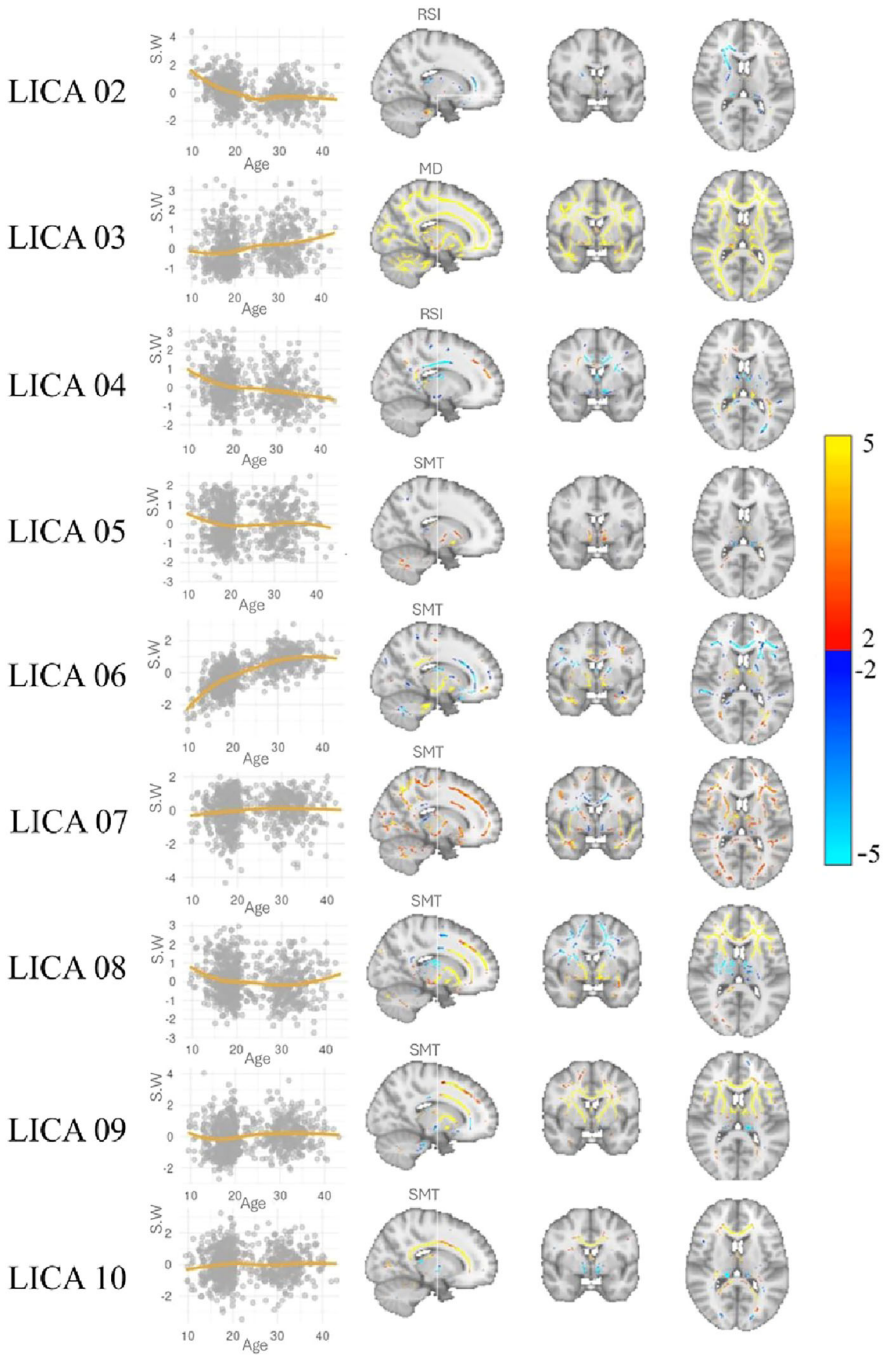
Weighted spatial maps from all LICA components and corresponding associations with age. For each component the model with the highest contribution is used (LICA 02 and 04: RSI, LICA 03: mean diffusivity (DTI), remaining components: SMT). All maps were thresholded with a minimum of 2 and maximum of 5 standard deviations (SD). Negative and positive weightings are shown in blue and red, respectively. LICA = linked independent component analysis, S.W = subject weights.

Ten LICA components were derived. One component (LICA 01) was mainly driven by a single individual, which was interpreted as a noise component, and excluded from further analyses. LICA 04 was largely driven by RSI neurite density (42%), and RSI fast fractional anisotropy (44%). The remaining components had contributions from 22 to all 24 metrics, suggesting broad contributions from a diverse set of metrics (see Figure S3). Figure 3 shows the associations between the LICA components and age, as well as an overview of the spatial maps for the remaining components after excluding LICA 01. The components had varying correlation with age, ranging from 0.03 to 0.7, with the strongest associations observed for LICA 06.

### 3.3 Associations Between dMRI LICA Components and CAPE

**FIGURE 4:**
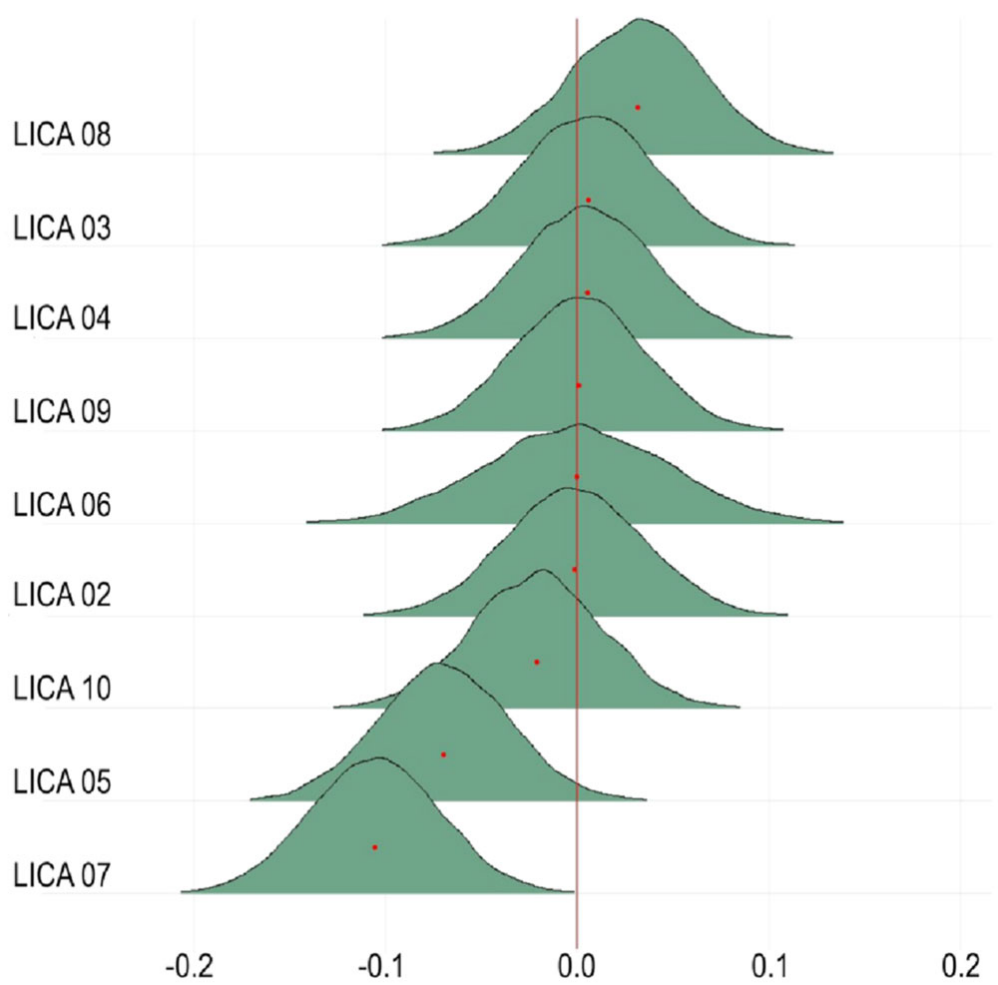
Posterior distributions of the association between total CAPE and the LICA components. LICA = linked Independent component analysis.

Figure 4 shows the posterior distributions reflecting the associations between the LICA components and the total CAPE score. The analysis provided moderate evidence for an association between CAPE and LICA 07 (BF = 0.15, *b* = −0.11). In addition, we found anecdotal evidence for no association for LICA 05 (BF = 1.9, *b* = −0.069) and strong evidence of no association for LICA 02 (BF = 14.18, *b* = −0.001), LICA 03 (BF = 13.83, *b* = 0.006), LICA 04 (BF = 13.71, *b* = 0.005), LICA 06 (BF = 10.35, *b* = −0.000), LICA 08 (BF = 9.69, *b* = 0.032), LICA 09 (BF = 15.31, *b* = 0.001), and LICA 10 (BF = 11.86, *b* = −0.02) (Summary stats are included in Table S2)

**FIGURE 5:**
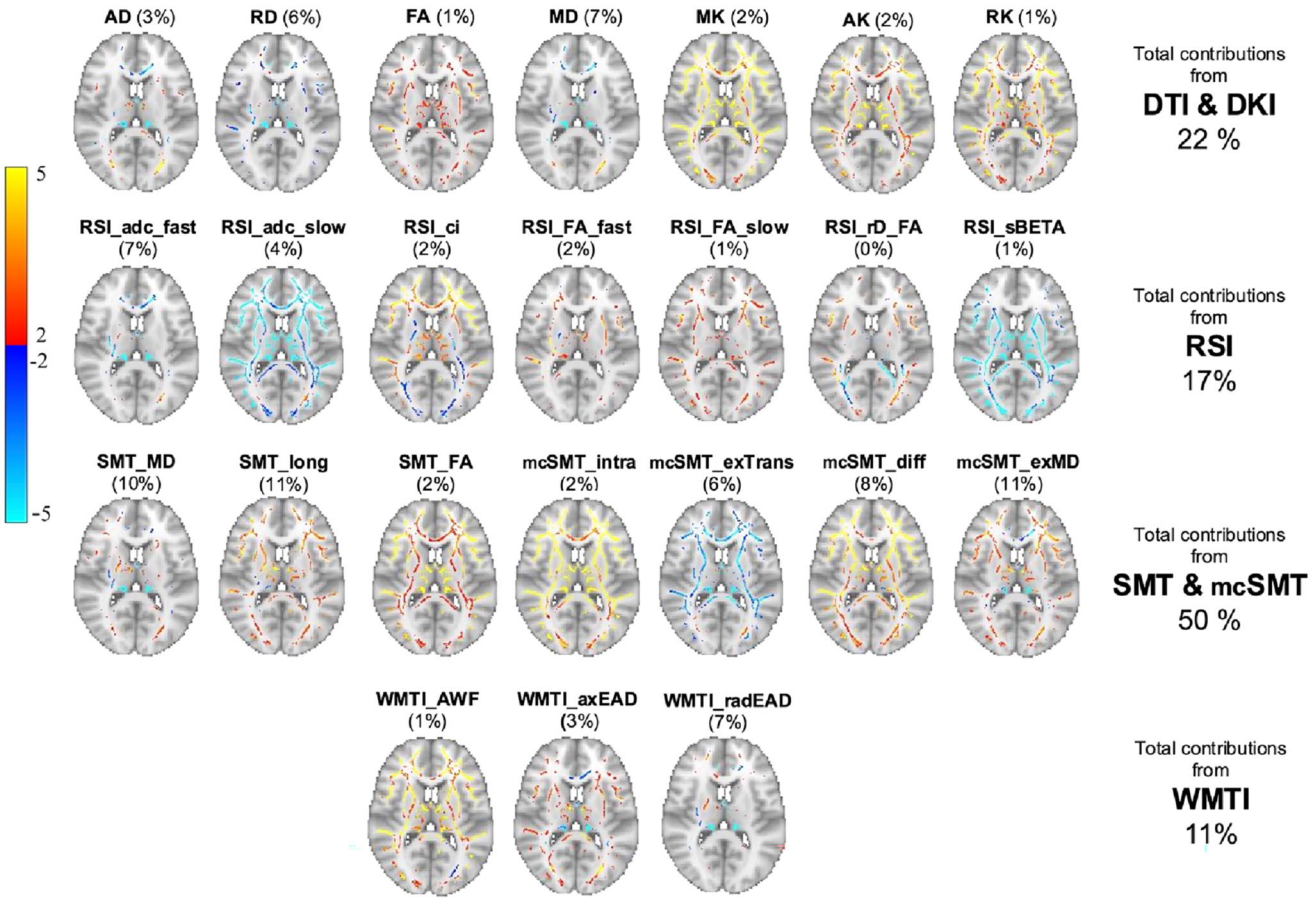
Weighted spatial maps for LICA 07 for all included modalities. For every modality the contribution into the LICA 07 component is given. To the left in each column is the total contributions from all the diffusion tensor imaging (DTI)/diffusion kurtosis imaging (DKI), restriction spectrum imaging (RSI), spherical mean technique (SMT)/multi‐compartment SMT (mcSMT) and white matter tract integrity (WMTI). The maps are overlayed on MNI152_T1_2 mm. Negative and positive standard deviations in the weightings are shown in blue and red, respectively. AD = axial diffusivity, RD = radial diffusivity, FA = fractional anisotropy, MD = medial diffusivity, MK = medial kurtosis, AK = axial kurtosis, RK = radial kurtosis, adc = apparent diffusion coefficient, ci = cellular index, rD_FA = Restricted diffusivity coefficient, sBETA = Neurite density, long = Longitudinal microscopic diffusivity, intra = Intra‐neurite volume fraction, exTrans = Extra‐neurite transverse microscopic diffusivity, diff = Intrinsic diffusivity, exMD = Extra‐neurite miscroscopic mean diffusivity, AWF = Axonal water fraction, axEAD = Axial extra‐axonal diffusivity, radEAD = Radial extra‐axonal diffusivty.

Figure 5 depicts the weighted spatial maps for LICA 07 for all included modalities. SMT models contributed 50% to the component, with the highest contribution from Longitudinal microscopic diffusivity (SMT_long_) and multi‐compartment extra‐neurite microscopic mean diffusivity (mcSMTexMD). Overall, LICA 07 comprises a complex spatial pattern with varying anatomical distributions between the contributing modalities. SMT_long_ implicates the inferior fronto‐occipital fasciculus, anterior thalamic radiation, and forceps minor, and mcSMTexMD additionally implicates the inferior longitudinal fasciculus. The FA map includes the superior longitudinal fasciculus shows and the MD maps show strongest weightings in the forceps major, followed by inferior fronto‐occipital and longitudinal fasciculus and the right part of the anterior thalamic radiation.

### 3.4 Associations Between dMRI LICA Components and CAPE Subscales

**FIGURE 6:**
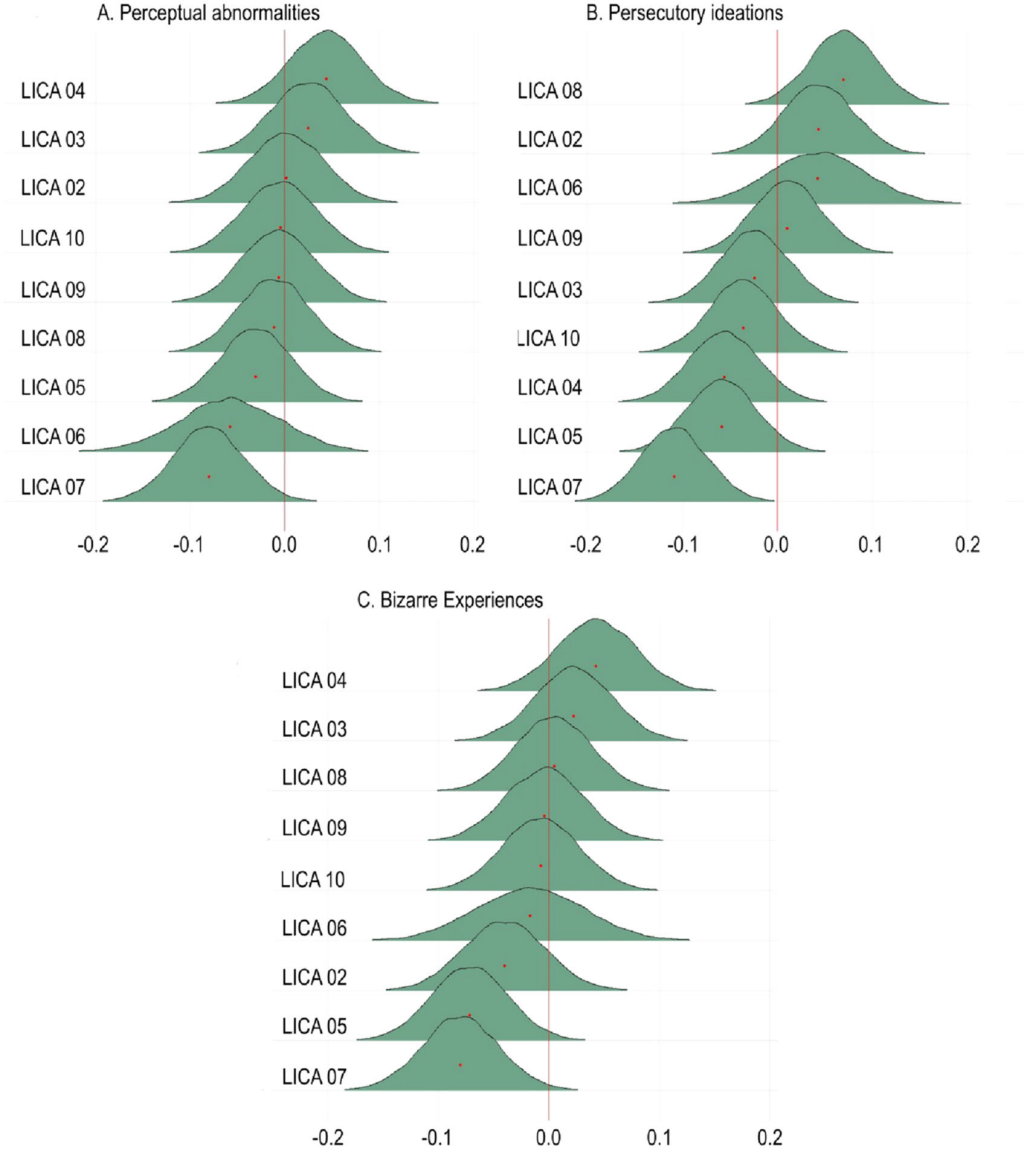
Associations between the CAPE subscales and LICA components.

Figure 6 shows the posterior distributions reflecting the associations between the three different subscales of CAPE and the LICA components. Overall, the results mimic the findings from the total CAPE score, with the strongest evidence of an association being moderate evidence for an association between LICA 07 and PIs (BF = 0.11, b = −0.11). In addition, the analysis revealed moderate evidence for no association between PIs and LICA 08 (BF = 2.08, *b* = 0.07), compared to the other CAPE scales where the evidence is strong for no association (PA: BF = 12.87, *b* = −0.01. BE: BF = 14.41, *b* = 0.01). (See Table S2 for further summary stats)

## 4 Discussion

PLE might not only be a risk factor for later psychotic disorders, but also a general susceptibility marker for mental distress in general. However, the link between PLE and white matter microstructure is currently unknown. By combining advanced dMRI and self‐reported symptom measures among 661 women aged 9–44 years we tested the hypothesis that regional patterns of white matter microstructural features are associated with PLE. Briefly, Bayesian analysis revealed that one component (LICA 07) from the LICA analysis was sensitive to PLE, with strongest evidence for the subscale reflecting PI. This subscale also had the highest mean scores across participants, and showed strong associations with other domains of psychopathology, supporting that PLE is associated with general psychopathology in this population‐based sample. Bayesian analysis revealed no evidence of an association between the other LICA components and PLE.

Metrics from all included diffusion models, including DTI, DKI, SMT, RSI, and WMTI, contributed to LICA 07, with the largest contributions from SMT longitudinal microscopic diffusivity (11%) and multicompartment SMT extra‐neurite microscopic mean diffusivity (11%). SMT longitudinal microscopic diffusivity is assumed to reflect diffusivities inside and outside the axons and dendrites, while SMT extra‐neurite microscopic mean diffusivity is related to extra‐axonal diffusivity and includes neurons, glial cells, and extracellular space (Kaden et al. 2016). Histological studies in animal models have shown that multi‐compartment SMT is sensitive to pathological tissue alterations (Kaden et al. 2016). Advanced diffusion metrics were key contributors to LICA 07 with 50 % contributions from SMT metrics. This indicates an added value of supplementing traditional diffusion metrics, such as DTI, with metrics obtained from more advanced models. In addition, the contribution from several modalities may implicate several tissue types and microstructural properties. The anatomical distribution of LICA 07 encompassed both positive and negative weightings in distributed regions, implicating major white matter pathways such as the inferior fronto–occipital fasciculus, anterior thalamic radiation, and forceps minor. Several previous studies have related disruption in fronto–thalamic white matter bundles to both early stage and chronic schizophrenia (Fryer et al. 2022; Moghaddam et al. 2024; Samartzis et al. 2014; Sussmann et al. 2009). Other studies have suggested more widespread group differences (Kelly et al. 2018), and previous work also reported associations between global FA and symptoms of psychosis both in youth and adults (Barth et al. 2023; Kjelkenes et al. 2022; Tønnesen et al. 2018). Studies examining white matter alterations and PLE in adolescence have reported mixed findings. While widespread abnormal WM microstructure in young adults with PLE have been reported (Barth et al. 2023; Drakesmith et al. 2016), others have only found associations between specifically visual hallucinations and white matter integrity in the Forceps Major (Schoorl et al. 2021). Another study in adolescents with PLE reported increased FA in striatal regions in proximity to the putamen, increased FA (in the right) and MD (in the left) uncinate fasciculus, and increased FA and decreased RD in parts of the right inferior fronto–occipital fasciculus (O'Hanlon et al. 2015). Our analysis implicated a complex spatial pattern, involving a range of anatomical regions and pathways. In line with previous studies, the inferior fronto–occipital fasciculus was among the top three loading for all modalities. We also see that the forceps major is implicated in modalities such as MD and RSI_adc_fast. We did not find a particular strong role of the uncinate fasciculus which has previously been implicated in both the PLE and schizophrenia literature (Barth et al. 2023; Kubicki et al. 2002; O'Hanlon et al. 2015; Singh et al. 2016)

Based on the concept that maturational processes during adolescence may be particularly relevant to the emergence of mental distress and disorders (Paus et al. 2008) we hypothesized that LICA components reflecting protracted neurodevelopment would be sensitive to PLE. Our analysis did not support this hypothesis, as the component showing strongest associations with CAPE (LICA 07) did not show any robust associations with age. It is likely that PLE and other mentally distressing symptoms is related to the development of the brain, but perhaps in other ways than a direct association with white matter microstructure captured in this cross‐sectional analysis.

It has been suggested that CAPE captures two overlapping phenomena; both a general psychopathology factor and a psychosis specific factor (Núñez et al. 2015). Our analysis revealed that individuals reporting high CAPE scores tended to report higher scores on measures of anxiety, depression, perceived stress, neuroticism, and low perceived social support from friends and family, and personality factors like conscientiousness and agreeableness. This supports that CAPE is sensitive to general psychopathology and not a specific marker for psychosis. This is consistent with a recent study in a Norwegian youth cohort where CAPE‐16 was found to correlate with current emotional symptoms (Birkenæs et al. 2024). The strongest evidence for an association between CAPE and the FLICA components was found for the PI subscale, comprising questions measuring paranoia. Previous studies have indicated that CAPE scores, and in particular the PI subscale, are consistently associated with depression and anxiety (Armando et al. 2010, Armando et al. 2012; Fusar‐Poli et al. 2012). In line with this, our analyses revealed that this subscale also showed the strongest correlations with the other domains of psychopathology.

Supporting previous studies, LICA revealed multimodal dMRI components with different sensitivity to age (Alnæs et al. 2018; Groves et al. 2012). LICA 06 showed a strong curvilinear relationship with age, with an apparent asymptote around the age of 35, possibly reflecting protracted white matter development well into young adulthood, in line with previous studies (Krogsrud et al. 2016; Lebel et al. 2012, 2017). The component encompassed a distinct pattern across several dMRI metrics, including models from DKI, SMT, WMTI, and RSI, with the largest contribution from SMT intra axonal diffusivity (15%). This has been found to reflect microscopic diffusion processes which enable the mapping of neurite density and compartment diffusivity, with the intra axonal model mainly capturing dendrites and axons (Kaden et al. 2016, By et al., 2018). The spatial map of LICA 06 encompassed a relatively global pattern, with the most negative loadings in the left anterior thalamic radiation, and a combination of positive and negative loadings in the right inferior fronto–occipital fasciculus and the left corticospinal tract. The pattern shared anatomical properties with LICA 07, although with negative loadings in the thalamic radiation and stronger involvement of the corticospinal tract. A previous LICA study of adolescence decomposed eight dMRI based white matter maps and identified a similar fronto–thalamic pattern including also the uncinate fasciculus and inferior parts of the fronto–occipital fasciculus (Alnæs et al. 2018). Here, loadings on the pattern were associated with general psychopathology and cognition (Alnæs et al. 2018). Although similar regions were represented in both LICA 06 and LICA 07, we only found associations with PLE in LICA 07. It is possible that LICA 06 was associated with other domains of psychopathology that is not captured through CAPE.

The existing literature on sex differences on the relationship between disruption in white matter and psychopathology is sparse. A large meta‐analysis in patients with schizophrenia and healthy controls revealed no significant group by sex interactions, but reported larger case‐control differences in FA among females compared to males when studying them separately (Kelly et al., 2018). Other reports have indicated sex differences in the associations between white matter dysconnectivity and psychopathology in the early course of schizophrenia, with smaller case‐control differences in females (Barth et al. 2023; Lang et al. 2018). Given the mixed findings it is possible that the current inclusion of female only may have revealed patterns that would otherwise be missed. Based on the reported associations between PLE and broader aspects such as depression and anxiety, which also show higher prevalence in women, the link between PLE and white matter features has an increased importance in women. We identified an association between a widely distributed white matter microstructural pattern and PLE in a sample of young to middle‐aged women. However, given that we have only included women, we cannot deduct whether this is a female specific pattern.

This study comes with several other limitations. First, the cross‐sectional design does not allow for delineation of individual developmental trajectories. Future longitudinal studies are needed to test the robustness of the associations as well as their temporal characteristics and relevance for future mental health outcomes. Similarly, a single time‐point was used for the questionnaires, which therefore only represents a snapshot of the current symptom–behavioral status. Next, while we consider the current focus on female mental health a considerable strength of this work, the results cannot be generalized to males. An additional limitation is that we have used a population‐based convenience sample that was generally high‐functioning. Future longitudinal studies will benefit from considering a broader spectrum of participants also including clinical groups.

In conclusion, our analysis implicated key commissural, association, and thalamocortical pathways in PLE among young female participants, with strongest effects for the PI subscale. The broad anatomical and modality contributions found in the LICA component showing associations with PLE indicate a pattern that is global and probably reflect multiple tissue types and microstructural properties.

## Author Contributions

**Rikka Kjelkenes**: conceptualization, data curation, formal analysis, investigation, methodology, project administration, resources, validation, visualization, writing–original draft. **Sara Fernandez‐Cabello**: formal analysis, methodology, visualization, writing–review and editing. **Irene Voldsbekk**: data curation, project administration, writing–review and editing. **Madelene Christin Holm Bukhari**: data curation, project administration, writing–review and editing. **Andreas Dahl**: data curation, writing ‐ review and editing. **Ingvild Sandø Lofthus**: data curation, project administration, writing–review and editing. **Henning Stople Rise**: data curation, project administration, writing–review and editing. **Christian K. Tamnes**: funding acquisition, writing–review and editing. **Ivan I. Maximov**: data curation, Methodology, Writing–review and editing. **Lars T. Westlye**: conceptualization, investigation, methodology, project administration, resources, supervision, writing–review and editing.

### Peer Review

The peer review history for this article is available at https://publons.com/publon/10.1002/brb3.70587

## Supporting information



Supplementary Materials.

## Data Availability

The data that support the findings of this study are available on request from the corresponding author. The data are not publicly available due to privacy or ethical restrictions.
