# Supplementary material for: Linking Psychotic‐Like Experiences and Brain White Matter Microstructure in Young Women
Source: Brain Behav. 2025 May 30;15(6):e70587. doi: 10.1002/brb3.70587 (PMC12123100; doi:10.1002/brb3.70587)
Supplement: Supplementary file 1 — Supplementary Materials. [file BRB3-15-e70587-s001.docx]

*Supplemental material*

**Supplemental figure S1.** Distribution of summary scores on measures of psychopathology and psychosocial adjustment.

| **Supplemental table S1.** Overview of diffusion metrics included in FSL’s LICA | | |
| --- | --- | --- |
| **Model** | **Metrics** | **Abbreviation used** |
| **DKI** (Diffusion kurtosis imaging) | Fractional anisotropy | **FA** |
|  | Mean diffusivity | **MD** |
|  | Axial diffusivity | **AD** |
|  | Radial diffusivity | **RD** |
|  | Axial kurtosis | **AK** |
|  | Mean kurtosis | **MK** |
|  | Radial kurtosis | **RK** |
| **RSI** (Restriction spectrum imaging) | Fast apparent diffusion coefficient | **RSI_ADC_fast** |
|  | Slow apparent diffusion coefficient | **RSI_ADC_slow** |
|  | Cellular index | **RSI_ci** |
|  | Fast fractional anisotropy | **RSI_FA_slow** |
|  | Slow fractional anisotropy | **RSI_FA_fast** |
|  | Restricted diffusivity coefficient | **RSI_rD_FA** |
|  | Neurite density | **RSI_sbeta** |
| **SMT** (Spherical mean technique) |  |  |
|  | Microscopic Mean diffusivity | **SMT_md** |
|  | Microscopic fractional anisotropy | **SMT_FA** |
|  | Longitudinal microscopic diffusivity | **SMT_long** |
| **Multi-compartment SMT** | Intra-neurite volume fraction | **SMT_intra** |
|  | Intrinsic diffusivity | **SMT_mc_diff** |
|  | Extra-neurite microscopic mean diffusivity | **SMT_extraMD** |
|  | Extra-neurite transverse microscopic diffusivity | **SMT_extraTrans** |
| **WMTI** (White matter tract integrity) | Axonal water fraction | **WMTI awf** |
|  | Axial extra-axonal diffusivity | **WMTI axEAD** |
|  | Radial extra-axonal diffusivity | **WMTI radEAD** |

**Supplemental figure S2**. Distribution of subject-weights for each independent component


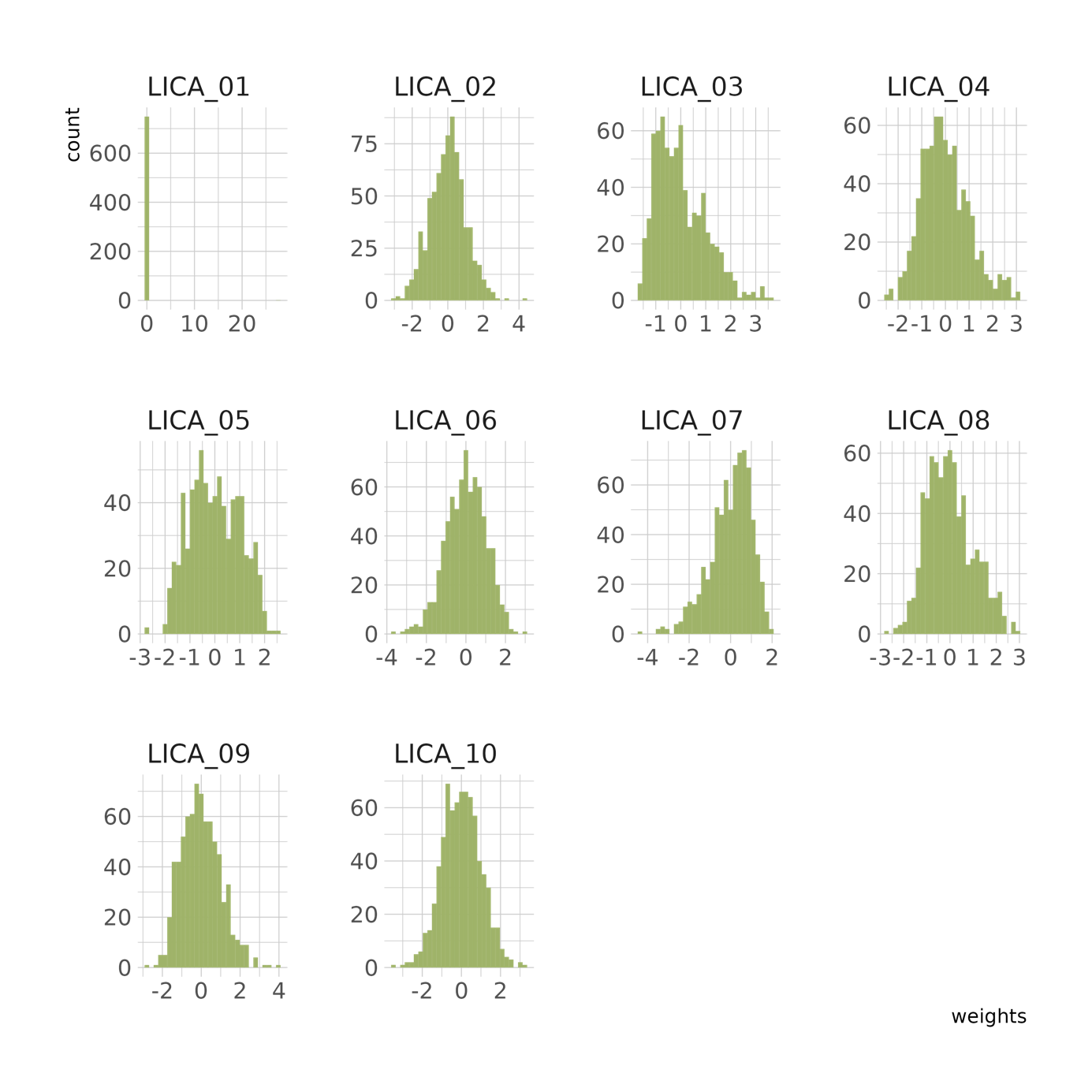


**Supplemental figure S3.** Overview of the relative weights of modalities on each of the independent components.


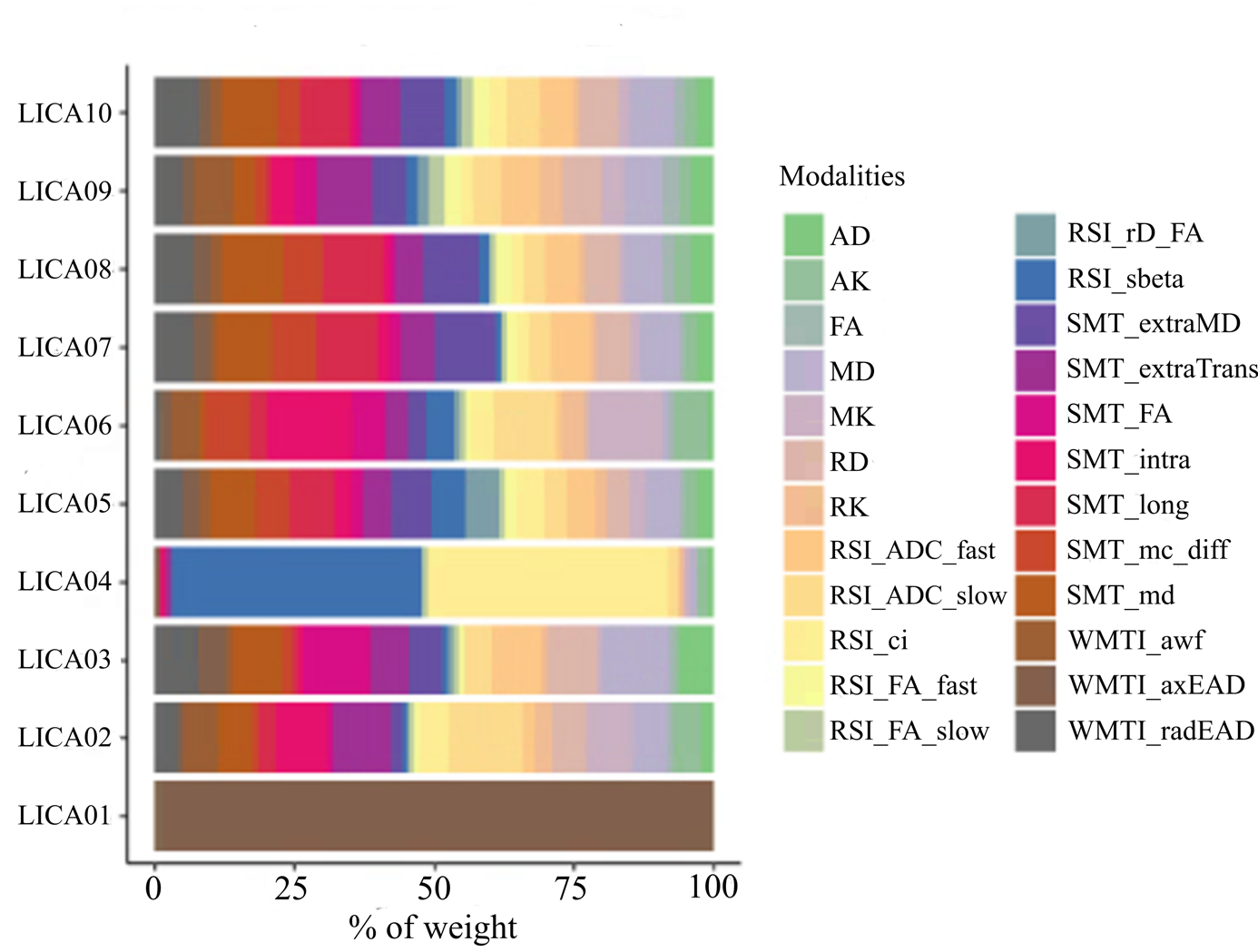


**Supplemental table S2.** Summary stats for Bayesian statistical analysis with the subject weights on the components from the linked independent component analysis and the various CAPE measures.

| *y* | *clin* | *estimate* | *lower95* | *upper95* | *p_higher_0* | *p_lower_0* | *evidence_0* | *BF_0* | *loo_elpd_diff* | *loo_se_diff* | *max_rhat* |
| --- | --- | --- | --- | --- | --- | --- | --- | --- | --- | --- | --- |
| CAPE_sum | IC002 | 0.00 | -0.07 | 0.07 | 0.48 | 0.52 | 14.18 | 0 | 0 | 0 | 1.00 |
| CAPE_sum | IC003 | 0.01 | -0.06 | 0.07 | 0.57 | 0.43 | 13.83 | 0 | 0 | 0 | 1.00 |
| CAPE_sum | IC004 | 0.00 | -0.06 | 0.08 | 0.55 | 0.45 | 13.71 | 0 | 0 | 0 | 1.00 |
| CAPE_sum | IC005 | -0.07 | -0.14 | 0.00 | 0.02 | 0.98 | 1.90 | 0 | 0 | 0 | 1.00 |
| CAPE_sum | IC006 | 0.00 | -0.09 | 0.10 | 0.53 | 0.47 | 10.35 | 0 | 0 | 0 | 1.00 |
| CAPE_sum | IC007 | -0.11 | -0.17 | -0.04 | 0.00 | 1.00 | 0.15 | 0 | 0 | 0 | 1.00 |
| CAPE_sum | IC008 | 0.03 | -0.03 | 0.10 | 0.83 | 0.17 | 9.69 | 0 | 0 | 0 | 1.00 |
| CAPE_sum | IC009 | 0.00 | -0.06 | 0.07 | 0.52 | 0.48 | 15.31 | 0 | 0 | 0 | 1.00 |
| CAPE_sum | IC010 | -0.02 | -0.09 | 0.05 | 0.28 | 0.72 | 11.86 | 0 | 0 | 0 | 1.00 |
| CAPE_pi | IC002 | 0.04 | -0.03 | 0.12 | 0.89 | 0.11 | 6.89 | 0 | 0 | 0 | 1.00 |
| CAPE_pi | IC003 | -0.02 | -0.09 | 0.05 | 0.25 | 0.75 | 10.76 | 0 | 0 | 0 | 1.00 |
| CAPE_pi | IC004 | -0.06 | -0.12 | 0.02 | 0.06 | 0.94 | 4.54 | 0 | 0 | 0 | 1.00 |
| CAPE_pi | IC005 | -0.06 | -0.13 | 0.01 | 0.05 | 0.95 | 3.97 | 0 | 0 | 0 | 1.00 |
| CAPE_pi | IC006 | 0.04 | -0.06 | 0.14 | 0.80 | 0.20 | 7.06 | 0 | 0 | 0 | 1.00 |
| CAPE_pi | IC007 | -0.11 | -0.18 | -0.04 | 0.00 | 1.00 | 0.11 | 0 | 0 | 0 | 1.00 |
| CAPE_pi | IC008 | 0.07 | 0.00 | 0.14 | 0.98 | 0.02 | 2.08 | 0 | 0 | 0 | 1.00 |
| CAPE_pi | IC009 | 0.01 | -0.06 | 0.08 | 0.61 | 0.39 | 13.37 | 0 | 0 | 0 | 1.00 |
| CAPE_pi | IC010 | -0.04 | -0.11 | 0.03 | 0.16 | 0.84 | 8.23 | 0 | 0 | 0 | 1.00 |
| CAPE_be | IC002 | -0.04 | -0.11 | 0.03 | 0.14 | 0.86 | 7.82 | 0 | 0 | 0 | 1.00 |
| CAPE_be | IC003 | 0.02 | -0.05 | 0.09 | 0.74 | 0.26 | 11.61 | 0 | 0 | 0 | 1.00 |
| CAPE_be | IC004 | 0.04 | -0.03 | 0.11 | 0.89 | 0.11 | 6.66 | 0 | 0 | 0 | 1.00 |
| CAPE_be | IC005 | -0.07 | -0.14 | 0.00 | 0.02 | 0.98 | 1.81 | 0 | 0 | 0 | 1.00 |
| CAPE_be | IC006 | -0.02 | -0.11 | 0.08 | 0.36 | 0.64 | 9.64 | 0 | 0 | 0 | 1.00 |
| CAPE_be | IC007 | -0.08 | -0.15 | -0.01 | 0.01 | 0.99 | 1.06 | 0 | 0 | 0 | 1.00 |
| CAPE_be | IC008 | 0.00 | -0.06 | 0.07 | 0.55 | 0.45 | 14.41 | 0 | 0 | 0 | 1.00 |
| CAPE_be | IC009 | 0.00 | -0.07 | 0.06 | 0.46 | 0.54 | 14.62 | 0 | 0 | 0 | 1.00 |
| CAPE_be | IC010 | -0.01 | -0.08 | 0.06 | 0.42 | 0.58 | 14.05 | 0 | 0 | 0 | 1.00 |
| CAPE_pa | IC002 | 0.00 | -0.08 | 0.08 | 0.51 | 0.49 | 12.94 | 0 | 0 | 0 | 1.00 |
| CAPE_pa | IC003 | 0.02 | -0.05 | 0.10 | 0.74 | 0.26 | 10.58 | 0 | 0 | 0 | 1.00 |
| CAPE_pa | IC004 | 0.04 | -0.03 | 0.12 | 0.87 | 0.13 | 7.09 | 0 | 0 | 0 | 1.00 |
| CAPE_pa | IC005 | -0.03 | -0.11 | 0.04 | 0.21 | 0.79 | 9.55 | 0 | 0 | 0 | 1.00 |
| CAPE_pa | IC006 | -0.06 | -0.16 | 0.05 | 0.14 | 0.86 | 5.61 | 0 | 0 | 0 | 1.00 |
| CAPE_pa | IC007 | -0.08 | -0.15 | -0.01 | 0.01 | 0.99 | 1.28 | 0 | 0 | 0 | 1.00 |
| CAPE_pa | IC008 | -0.01 | -0.09 | 0.06 | 0.39 | 0.61 | 12.87 | 0 | 0 | 0 | 1.00 |
| CAPE_pa | IC009 | -0.01 | -0.08 | 0.07 | 0.43 | 0.57 | 13.01 | 0 | 0 | 0 | 1.00 |
| CAPE_pa | IC010 | 0.00 | -0.08 | 0.07 | 0.45 | 0.55 | 12.32 | 0 | 0 | 0 | 1.00 |
